# Referral of patients to diabetes prevention programmes from community campaigns and general practices: mixed-method evaluation using the RE-AIM framework and Normalisation Process Theory

**DOI:** 10.1186/s12913-019-4139-5

**Published:** 2019-05-22

**Authors:** Sarah Knowles, Sarah Cotterill, Nia Coupe, Michael Spence

**Affiliations:** 1 NIHR Greater Manchester Collaboration for Leadership in Applied Health Research and Care, Alliance Manchester Business School, University of Manchester, Manchester, UK; 2 Centre for Biostatistics, School of Health Sciences, University of Manchester, Manchester Academic Health Science Centre, Manchester, UK; 3 Manchester Centre for Health Psychology, School of Health Sciences, University of Manchester, Manchester, UK; 4 NIHR Greater Manchester Collaboration for Leadership in Applied Health Research and Care, Salford Royal NHS Foundation Trust, Salford, UK

**Keywords:** Diabetes prevention, RE-AIM, Normalisation process theory, Primary care, Community

## Abstract

**Background:**

Each year around 5–10% of people with non-diabetic hyperglycaemia will develop type 2 diabetes mellitus. Diabetes prevention is a national and global public health concern. Diabetes Prevention Programmes, which seek to identify at-risk individuals and support entry to health improvement initiatives, recognise that enhanced identification and referral of at-risk individuals is required within primary care and beyond, through community-focused prevention approaches. We report an evaluation of a demonstrator site for the NHS Diabetes Prevention Programme in the UK, which piloted an enhanced Primary Care referral programme (sampling from patients identified as at-risk from general practice databases) and a Community identification programme (sampling from the general population through opportunistic identification in community locations) in an effort to maximise participation in prevention services.

**Methods:**

We used mixed-methods evaluation to assess the impact of the two referral routes on participation in the Diabetes Prevention Programmes in line with the RE-AIM Framework (Reach, Effectiveness, Adoption, Implementation and Maintenance). Individual level patient data was descriptively analysed to assess identifications and eligible referrals made in each route. Semi-structured interviews conducted with referral staff and key stakeholders were analysed using thematic analysis and informed by Normalisation Process Theory.

**Results:**

The nurse facilitated primary care referral route provided 88% of all referrals to the telephone DPP, compared to the community referral route which provided 5%, and the proportion joining the programme was higher among primary care referrals (45%) than community referrals (22%), and retention rates were higher (73% compared to 50%). The nurse-facilitated route integrated more easily into existing clinical processes. The community programme was impeded by a lack of collaborative inter-agency working which obscured the intended focus on high-risk populations despite conversion rates (numbers identified at risk who entered prevention programmes) being highest in areas of high deprivation.

**Conclusions:**

The study demonstrates the interaction of components, with effective Adoption and Implementation necessary to support Reach. The NPT analysis demonstrated the importance of consensus around not only the need for such programmes but agreement on how they can be delivered. Future programmes should support inter-agency communication and collaboration, and focus identification efforts on areas of high-risk.

**Electronic supplementary material:**

The online version of this article (10.1186/s12913-019-4139-5) contains supplementary material, which is available to authorized users.

## Background

Non-diabetic hyperglycaemia is a condition where blood glucose levels are raised above normal levels, but are not high enough for a diagnosis of type 2 diabetes mellitus (T2DM). Without treatment 5–10% of people with NDH will go on to develop T2DM every year [1]. Government data indicates that 3.8 million people (9% of population) in England aged over 16 had diabetes in 2015, and 90% of those have T2DM. T2DM can lead to an increased risk of developing other cardiovascular health problems [2] and poses a considerable economic burden. For example, In the UK, a cost of £10billion per year to the NHS is estimated, which would equate to around 9 % of the total NHS budget [3]. Such costs and consequences indicate that diabetes prevention is a priority concern for public health.

In the UK the risk of T2DM rises with age, is slightly higher in men than women, and is substantially higher among people from South Asian and Black communities. Deprivation is strongly associated with obesity, inactivity, poor diet, smoking and poorly controlled blood pressure, all of which are linked to T2DM risk [3]. Preventative treatments, for example changes in lifestyle and behaviour which lead to weight loss, are known to be effective. This includes for example changes in diet and increasing physical activity, which have been shown to decrease the risk of non-diabetic hyperglycaemia developing into T2DM by 50% [4, 5]. However, the asymptomatic nature of nondiabetic hyperglycaemia combined with a lack of awareness of diabetes symptoms can lead to under-diagnosis. Opportunities to identify those at-risk and offer effective preventative treatments are often missed [6].

In response, Diabetes Prevention Programmes (DPP) have been developed and implemented worldwide [7, 8], with the aim of reducing the incidence of T2DM through targeting those considered at risk of developing it, and encouraging them to modify their lifestyle behaviours. Large randomised controlled trials of these ‘screen and treat’ programmes have demonstrated that through a relatively modest weight loss of 5–7%, such lifestyle interventions can reduce the risk of developing T2DM by up to 58% [9]. This illustrates the importance of weight loss in the prevention of T2DM, as the risk of developing it in these studies reduced by 16% for each kilogram of weight lost [7]. This also highlights the role of obesity in the rise of T2DM, and supports the targeting of weight reduction for the prevention of the disease [4]. Although such results are promising, notably these findings are from controlled trials and pragmatic evaluation is still in its infancy.

Healthier You: The NHS Diabetes Prevention Programme (NHS DPP) is being implemented in England with the intention to offer 100,000 referrals per year by 2020. The NHS DPP offers tailored, personalised help to people at risk of diabetes (T2DM), aiming to reduce the risk of type 2 diabetes through education on healthy eating and lifestyle; help to lose weight and bespoke physical exercise programmes [2]. People with non-diabetic hyperglycaemia will be identified through NHS Health Checks and registers in primary care.

During 2015–2016 seven demonstrator sites were commissioned to test innovative approaches to programme delivery, with a view to shaping the English NHS DPP programme. One of the demonstrator sites was in an urban setting in a city in North West England. Rates of diabetes, deprivation and obesity were all above the average for England. The demonstration site adopted a DPP service, available to eligible patients (eligibility assessed by the Leicester Diabetes Risk score) which involved:

- **A tailored exercise programme** – A structured 8 week programme delivered in community health centres and other local venues by exercise trainers employed by local government,
- **A telephone service** – The core elements of the phone calls involved educational messages, goal setting and action planning as part of an 8 telephone call programme over a 9 month period, which was loosely based on motivational interviewing [10, 11]. The telephone service was based in a local hospital, overseen by a diabetes clinical team and delivered by non-clinical health advisors, using a range of scripts to guide conversations with patients.
  Eligible patients could choose to attend one or both programmes.

One crucial aspect of public health evaluation is the need for explicitly consider the ‘reach’ of the intervention, meaning the number of eligible patients that an intervention is delivered to, and explicitly considering how programmes identify and refer those who could benefit from the intervention. This can be neglected in, for example, randomised controlled trials, as eligibility is addressed in terms of inclusion or exclusion from those identified to participate trial, rather than considering broader reach in population terms (how effective the programme is at identifying and recruiting eligible patients). The NHS DPP programme seeks to specifically improve identification and referral of at-risk individuals, evaluating the reach of innovative strategies targeted at people who may be neglected by traditional programmes. Primary care or community campaigns targeted towards the populations most at risk of T2DM may prove fruitful in identifying people for referral to a DPP. The demonstrator site had previously relied on referrals from GP practices and had experience of difficulty in identifying sufficient people with NDH to refer into the local DPP programmes. They adopted two innovative referral methods in an attempt to reach a broad selection of the local at-risk population:

1. **Community referral** – Two public health associated agencies proactively screened and tested the general public at a number of locations across the conurbation, to find people who were at risk of T2DM.
2. **Nurse facilitated primary care referral** - A nurse worked with local GP practices, to search through patient records to identify people who were at risk of T2DM. The nurse also held specialist clinics to promote and refer suitable patients into the intervention.

The aim of the study was to evaluate the impact of these two novel interventions on the identification and referral of people into diabetes prevention service, to consider their impact on Reach, and also assess their Adoption, Implementation and Maintenance. We therefore aimed to evaluate the effectiveness of the Reach of these interventions, but have deliberately not described this as ‘Effectiveness’ in the RE-AIM model to avoid confusion over terms, given that Effectiveness in the RE-AIM literature is typically used to describe impact on clinical outcomes.

## The RE-AIM framework

The RE-AIM framework has been applied to a variety of public health problems, health promotion interventions and chronic disease topics [12]. The framework promotes a comprehensive analysis of factors affecting internal and external validity, to aid in evaluation of public health programmes and also translation of findings to other settings [13]. In the present study we aimed to use the framework to understand the impact of two referral methods for increasing recruitment to a diabetes prevention programme. The framework was employed to support a synthesis of qualitative and quantitative data that evaluated both process and outcomes, and to enable us to make explicit recommendations regarding delivery of the interventions elsewhere to inform a national delivery programme. The RE-AIM dimensions and corresponding research questions are presented in Table 1.

**Table 1:** RE-AIM dimensions and corresponding questions

RE-AIM Dimension	RE-AIM questions	Study research question
Reach	What percentage of potentially eligible participants are recruited, and how representative are they?	How many people are judged to be at risk for IGR, and how many were recruited? How representative are those recruited of the local population? How many people were recruited from high-risk populations? Which treatments did participants enter? Which referral route was most effective in recruitment of eligible participants?
Effectiveness	What impact did the programme have on targeted outcomes?	*Clinical outcomes for individuals were not evaluated in this study.*
Adoption	Which setting and intervention agents were involved and how representative are they?	What features of the participating services were considered crucial to delivery? Did engagement vary across the different services? What characteristics of services are required if the intervention was to be adopted elsewhere?
Implementation	Was there fidelity to the intervention, and what local adaptation occurred?	What were the barriers and facilitators to delivering the intervention as planned? How did contextual factors impact on delivery? How was the intervention tailored to different populations? Was delivery adapted over time, and if so why?
Maintenance	Individual level – extent to which behaviours are maintained long term; Institutional level – extent to which programme is sustained over time.	Individual: which factors are associated with adherence to treatment – does referral route impact on this? Institutional: What factors will impact on continuation of the programme? What resources are considered necessary to improve delivery?

A review of diabetes self-management education interventions which employed the RE-AIM framework recommended more formal evaluation of the process of both implementation and adaptation (with particular attention needed to adaptation to under-served populations), and also called for greater attention to the community context of such work [14]. Qualitative methods have been underutilized in terms of explaining or contributing to RE-AIM dimensions [12]. We employed Normalisation Process Theory (NPT) to add greater depth of understanding to the qualitative findings on Adoption, Implementation and Maintenance by mapping the constructs of NPT to the research questions addressed by the qualitative part of the study. NPT is a model of the collective action required to integrate a new way of working into routine service [15, 16].

### NPT and RE-AIM constructs and questions

We integrated the NPT and RE-AIM constructs to evaluate the following aspects of the programmes:

#### **Adoption:** how engaged are providers? What factors impact on their engagement?

***Coherence:*** Sense-making and shared purpose – do participants agree on the need for the intervention and the expected benefits?

***Cognitive participation:*** The relational work that is undertaken - are key people involved in driving the work forward? Is there collective agreement on who should be involved and their roles in the work?

#### **Implementation:** to what extent was the intervention implemented as planned? What were the barriers to implementation?

***Collective action:*** Interactions and relationships between those involved in the work – are there effective mechanisms for working together? Do those involved have confidence in each other and agreement about processes and division of labour?

***Reflexive monitoring:*** Assessment and understanding - Is there shared appraisal of the work, consensus on worth and collective reconfiguration of practices?

## Methods

The two referral methods which were evaluated consisted of:

1. **Community referral** – Two agencies were commissioned to engage with the local community and make referrals to the DPP: a local authority health improvement team and a voluntary sector organisation, both with previous experience of similar public health promotion campaigns. They set out to raise awareness of T2DM and conduct initial diabetes risk scoring assessments, using the Leicester Diabetes Risk Score. HbA1c finger prick blood tests were subsequently offered to people who scored medium-high on the diabetes risk score. It was hoped this community approach would be a way of targeting a broader spectrum of people at risk of diabetes than primary care could find, including those from ethnic minorities and deprived areas where the populations are most at risk. The two agencies had experience of working within the local communities, with one of the agencies relying on neighbourhood champions within the community to assist with engagement.
2. **Nurse facilitated primary care referral** – A qualified nurse, with previous involvement and experience of the local telephone based service attended a number (*n* = 16) of GP practices within the city. The nurse searched through electronic health records for Read codes that would indicate people at risk of T2DM and work with the GP Practice to invite these patients to group clinics, held by the nurse within the GP Practice to discuss referral to the NHS DPP.

### Quantitative Evaluation

#### Design

Individual patient-level analysis of routine data relating to identification and referral of patients to the DPP between October 2015 and March 2016, collected by three service providers.

#### Population and data sources

Individual level patient data was recorded by three service providers. The local authority recorded data on all people who completed diabetes risk scores in community settings during the study period. Data collection included demographic information, diabetes risk score, HbA1c, weight and details of any DPP referrals. The hospital provided data on everyone who was referred to the telephone DPP during the study period and the local authority provided similar data on referrals to the exercise DPP. Both services recorded the referral source, take-up of the DPP service and number of sessions attended. The datasets were anonymised by the data providers and passed to the research team.

#### Analysis

There was no common personal identifier in use, so the data were analysed as three separate datasets using Excel and Stata 14. Data was summarised using descriptive statistics; number (percentage) for categorical variables and mean (SD) for numeric variables. Results for all three data sources are presented using the Re-Aim framework. Reach includes the numbers who were approached, tested and referred, by referral route (community or GP) and whether the community campaign was successful in targeting those at highest risk of diabetes. We present an analysis of the community referral route in terms of factors associated with higher risk: age, gender, ethnicity and deprivation [17], comparing those referred with the local population. Unfortunately, comparison data on those referred from primary care was not available. We also assessed the number of people referred to the two DPPs, as a proportion of those who were approached, by referral route. Finally, maintenance considers retention of people in the two DPPs. ‘Effectiveness’ is not assessed as it typically refers to the impact of a programme on clinical outcomes, which are not reported here. Rather, the present study was concerned with the effectiveness of the referral methods, and is therefore reported as ‘Reach’.

### Qualitative Evaluation

#### Design

Qualitative semi structured interviews and focus groups were conducted with a purposively selected sample of 32 participants. In total, we conducted 21 individual interviews and 4 focus groups (3 groups with 3 participants and 1 group with 2 participants).

#### Population and data collection

The sample was identified through liaison with service leads in late 2015, to provide representation from all stages of the pathway. It was designed to include decision makers/service leads as well as frontline workers involved in the delivery of the services and staff working in GP practices. A breakdown of the participant numbers is detailed in Table 2. All interviews and focus groups took place between February and June 2016.

**Table 2:** Breakdown of interview and focus group participants

Community referral service	16
Decision makers and service leads	8
Primary care	6
Exercise	2
Complete sample size	32

The topic guides (Additional file 1) were developed based on the evaluation aims and through discussion with key stakeholders including commissioners and service managers, to identify important issues to explore. Participants provided written consent prior to taking part. All interviews were recorded and transcribed verbatim, and supplemented by field notes taken by the interviewers. Transcription was performed by an independent service.

#### Analysis

We analysed the data using the Framework approach. Data were entered into NVivo for organisation and coding. The framework was derived explicitly from the topic guide and consisted of thematic categories of:

1. Experiences of implementation of the referral process, including inter-organisational working and data sharing.
2. Acceptability to staff themselves
3. Perceived acceptability for the public,
4. Perceived benefits
5. Perceived risks/challenges,
6. Suggestions for improvement.

Initially, the 3 researchers conducting the analysis each openly coded a subset of 3–5 transcripts. This enabled familiarisation with the data, unrestricted by the a priori framework, including analysis by a researcher who was external to the evaluation team and had not conducted any interviews (in order to include the perspective of someone outside the study), to check whether there were findings which did not fit into the original categories. All researchers agreed that the framework categories adequately reflected the prominent issues in the data, and no additional categories were needed. The framework was then applied by the 3 researchers independently to the entire sample (with each transcript coded by 2 researchers). A consensus meeting was then held with the wider study team (including all authors) to agree on a final interpretation of the data and the core themes (these are represented in Column 3 of Tables 3 and 4).

**Table 3:** Thematic Analysis of Primary Care data

Topic	Exemplar Data	Initial interpretation & analysis	Interpretation with NPT Constructs
1. Adoption: engagement of providers			
1a) Coherence: consensus, agreement and congruence around shared purpose	“*GPs [General Practitioners] are interested in health promotion and disease prevention, but actually they’ve already got a load of stuff to do with people who are already ill.”* Primary focus group 1 *“I think we’re very restricted time-wise because of staffing to sit down with patients to impart that information and utilising the services that are setup.”* Primary 3	Value of prevention recognised but accompanied by perception that practices themselves are under- resourced to deal with this.	High *coherence*, with all participants agreeing on the need for a prevention programme with patients at risk of diabetes, and agreement that existing practice resource was insufficient.
1b) Cognitive participation: roles and relationships	*“You need to be a healthcare professional, yeah … You’ve got to interpret the blood results, look at the patient records to see if they’re suitable.”* Lead 5 *“Having somebody that knew about diabetes … and able to make decisions at a higher level. You need that gravitas of someone who’s clinically trained. The patients respect that more.”* Primary focus group 1 “*I put together flowcharts for our surgery about what to do around blood sugar, I adapted the stuff that the nurse facilitator had originally sent me and I did a flowchart for staff on how to refer so I’ve done quite a lot of work … It’s taken months of implementing*.” Primary 3	The support of the NF (Nurse Facilitator) was viewed by practices as providing additional full capacity to co-ordinate and deliver referrals (as opposed to supporting the practices to do this themselves). Perceived as essential that the role was for a Nurse, who could interact with the clinical systems and with patients in a clinical capacity. Practices which did not receive full support reflect on complexity and burden of referral process.	*Cognitive participation* was straight-forward given that consensus was that the NF needed to act independently rather than requiring additional work by the practice staff. There was also agreement that the nurse facilitator was the appropriate role, as the person offering support needed to be clinically trained. In those practices where the full support of the NF was not provided, practices instead reflected on the burden created by referral, again demonstrating that *cognitive participation* for these participants centred around the difficulty of practices themselves trying to do the work and therefore needing full support.
2. Implementation and barriers to implementation			
2a) Collective action: relationships and confidence in each other	“*A lot of [GPs] felt that the education they already gave them [patients] was adequate … so their question was why should we refer in?... But when you then explain how you go into things in great detail [in the telephone service] and what we actually do, they could see what’s going on*.” Lead 5	The NF role as part of the telephone service increased understanding of the service.	The model of NF was simple to implement in comparison to the community route given that a need for *collective action* was largely avoided – the role of the NF in conducting the work in practices and liaising directly into the telephone service meant the interactions were simplified. The NF’s dual role as part of the telephone service itself further embedded trust in the process by being able to directly communicate value to GPs.
2b) Reflexive monitoring: extent to which there is a shared understanding about the intervention.	*“It’s certainly built up relationships. I still get emails now from doctors in different practices and practice nurses, so they’re now aware of the service which before they may not have been... … they now know that they’ve got somebody to contact if they need the help … I think once you’ve got that link they will be more receptive to referring in and the services and helping the patients.”* Lead 5 *“I’d refer to our Health Care Assistant [HCA] … they’d get a face-to-face with a HCA. I could understand if I was getting some diabetic input … If it’s just for a chat on the phone then I wouldn’t refer. If it was just to a health care person then I’d do that in-house.” Primary focus group 1*	Participants again reflected on the value of the NF being integrated with the telephone service itself, which provided reassurance and an accessible way to clarify issues regarding referral and the service. Possible changes to the telephone service intervention itself (removing a first contact with a Diabetes Specialist Nurse) may threaten the evaluation of the service as useful, and consequently the need for referral.	*Reflexive monitoring* was supported by the NF being the person performing the referrals and also part of the telephone service itself, providing a clear means for staff to understand the value of the service. However, this may be undermined if primary care staff felt that patients would not benefit from additional specialist input, demonstrating how *congruence* (perceived value) can be reflectively reassessed and may decrease *cognitive participation* and *collective action*.

**Table 4:** Thematic Analysis of Community Referral data

Topic	Exemplar Data	Initial interpretation & analysis	Interpretation with NPT Constructs
1. Adoption: engagement of providers			
1a) Coherence: consensus, agreement and congruence around shared purpose	*“[The CCG – Clinical Commissioning Group] I think are really supportive of community based interventions. They appreciate that it brings something different to the table.”* 0I9 (Community Service Lead) *“The intervention we’re being asked to signpost into is an intervention that’s based in secondary care services at the hospital, and yet these people aren’t ill...So the whole fundamental way they think about it is to treat people in a clinical way, and it goes against the ethos and the way that we would work”* 0030 (Community referral provider focus group) *“It’s really frustrating for our staff and demotivating when they’re seeing budget cuts continually in this service and then they’re seeing another service being commissioned to do what they were doing*” 0030 (Community referral provider focus group)	Buy-in to the need for community referrals at all levels. Both Community agencies referred to similar benefits in comparison to clinical referrals (proactive, raising awareness, being more approachable) and similar requirements (flexibility, the importance of local knowledge) Tensions between the community and clinical services regarding their different approaches, and tensions between the two community services in the context of limited funding, Unclear on value of both community services working together, what this added.	There was *coherence* from all participants (frontline workers, managers, and commissioners) for the need for community referral as a different way of working and the likely benefits. However, there was a lack of *coherence* regarding how this would be achieved and the value of the two community services working together.
1b) Cognitive participation: roles and relationships	*“I think communication and an understanding of peoples roles, and a understanding of how people fit in, into the whole process, I think that was our kind of key. And some people maybe had a misunderstanding of what the role was … I would of liked … the expectations in place and then start, … getting expectations drawn up between the different parties”* 033 (Community referral provider co-ordinator) *“I didn’t get...personally didn’t get my head round Care Call until much later into the pilot. How does it work? How does that link up with the case finding?”* 019 (Community referral provider service lead)	Lack of clarity about how the collaboration would work in practice and how the different services were expected to work together.	*Cognitive participation* was problematic. The services involved did not share understanding about the work to be done (both the collaboration between the community services and the interaction between the community services and Care Call) and the processes required, and in the early stage of the pilot had not been brought together to collectively resolve the issues
2. Implementation and barriers to implementation			
2a) Collective action: relationships and confidence in each other	*“It’s really fragmented and broken down. There’s loads of different people involved in it and nobody knows what anybody else is doing.”* 0030 (Community referral provider leads focus group) *“One of the problems has been communication …*. I *think sometimes when you’ve got a service like [Care Call]that’s very clinical and it’s almost quite a closed system and they know the referrals they’re getting through is very clean, I don’t think they understand how difficult it is for us to actually case find in the community.”* 0030 (Community referral provider leads focus group) *“After speaking to [leads] they just said to me get out as much as you can and get as many people as you can … the fact of working with [the other service] and them referring in, I don’t really get where all that’s working together.”* (042 Community referral provider frontline worker) *“While we are trying to target sometimes you don’t try to target too much. You’ve got to hit the numbers*.” (028 Community referral provider frontline worker)	The lack of agreement on how to work together meant that provision was fragmented rather than collaborative, and divisions between the services were maintained. This had 2 impacts on the referral process itself 1. Care Call were unprepared for the different referrals received from the community services. The community services felt that Care Call did not understand the work they were doing and had unrealistic expectations about what could be provided (for example, NHS Numbers) which led to delays in referrals being processed. 2. The focus on targeting was obscured by a focus on each service trying to “make up the numbers” rather than working effectively to co-ordinate the work required.	*Collective action* was not achieved – the division of labour and mechanisms for working together were unclear. The services did not have confidence in each other which led to delays and meant that the focus on targeting high-risk areas was partly abandoned.
2b) Reflexive monitoring: extent to which there is a shared understanding about the intervention.	*“What’s been useful is the meetings that we’re having with [clinical service] around this project because we’ve relayed that information and we’ve actually had people from there attend and we’ve been able to put those to them and say, you know, we want the feedback.”* (Community referral provider frontline focus group) *“When we’re [community agencies] working together it’s really important that we know what they want but it’s also very important for them to tell us what they want... So communication is, we’ve learnt a lot from this about how different we work together”* (Community referral provider frontline focus group)	Over the course of the pilot, the services all made efforts to improve their communication and understand each others’ roles. This led to an appreciation of the need to collectively understand the issues from each others perspective in order to resolve them.	*Reflexive monitoring* emerged as key over the course of the pilot, with services needing to come together directly to share their experiences and preferred ways of working, and revise processes where required, including establishing mechanisms for further feedback. The learning in terms of understanding each service and how to work together was highly valued but took time to develop.

We subsequently applied the NPT constructs of coherence, cognitive participation, collective action and reflexive monitoring through selective coding of data in the original framework (Column 4 of Tables 3 and 4). The rationale for this secondary coding was two-fold: firstly, to consider the generalisability of the specific results beyond this context by employing an established conceptual framework. Secondly, to use the framework to more deeply explore the core themes, reflecting on mechanisms of action underpinning the overall findings, and therefore link the qualitative and quantitative analyses to produce a more complete explanation. A consensus meeting was held with all team members, including the original coders and all who had conducted interviews, to agree on interpretation and application of the NPT framework to the original data and again consider whether any key issues had been excluded.

It was agreed that the constructs had immediate face validity regarding the core themes, and selective coding into the framework was used to develop further insights into adoption and implementation.

## Results

### Reach – comparison of community and primary care identification

#### How many people are judged to be at risk for IGR, and how many were recruited?

- *Community.*
  Staff from the community agencies completed a diabetes risk score [18] for 1162 people. The diabetes risk score had four categories: low risk, increased risk, medium risk, and high risk of developing T2DM. Among the 1162, 791 people (68%) scored medium or high, suggesting that they should be offered a blood test (Table 5). HbA1c blood tests were completed for 746 people. Of these, 740 (99%) were for people who were medium or high risk and only 6 (1%) were for people at low/increased risk (Table 5). There were 51 people at medium or high risk who were not given a blood test, and no reasons were given. Among the 746 people who had blood tests done, 71 (10%) had NDH and 18 (2%) had diabetes. The remainder were normal results. The number of people who were referred to a diabetes prevention programme was 66 people (63 NDH, 1 diabetes and 2 not tested), 6% of those who completed a diabetes risk score.
- *Primary care.*
  In total 883 people were referred from 46 primary care practices to the two diabetes prevention services. A nurse facilitator searched electronic records to identify suitable patients in 16 practices, and held clinics in 13 of those practices. Of these 883 primary care referrals, 774 (88%) came from 16 practices where there had been some engagement with a nurse facilitator and 109 (12%) came from the other 30 practices.

**Table 5:** Community agencies - diabetes risk scores and HbA1c blood tests

Diabetes risk score category	Number with risk score (% of total)	Number given blood tests (% of those with risk score)
No risk score done	1 (0%)	0 (0%)
Low/increased risk	371 (32%)	6 (2%)
Medium risk	517 (44%)	468 (90%)
High risk	274 (24%)	272 (99%)
Total	*n* = 1163	*n* = 746 (64%)

#### How representative are those recruited of the local population?

The motivation for introducing the community approach was to expand the number of referrals, over and above those identified in primary care, and to find individuals most at risk of diabetes.

- *Age:* Completion of risk scores and referrals to the DPP were far higher among older people, with almost half of referrals (46%) aged over 70.
- *Gender:* Both the risk scores and the referrals show a large imbalance of women, who made up 65% of all risk scores and 68% of all referrals.
- *Ethnicity:* 7% of those who completed risk scores were Black and Minority Ethnic (BME), and 16% of referrals were BME, compared to the the proportion of BME in the local population (10%).
- *Deprivation:* r The mean number of risk scores completed per ward was 41 in the five most deprived wards and 75 in the five most affluent wards.

#### Diabetes prevalence

The research population had a high rate of T2DM, with 15 out of 19 wards having a diabetes prevalence that exceeded the English prevalence rate of 5%. T2DM prevalence varied across the city, ranging from 4.4 to 7.9%. Of the five areas with a prevalence of over 7%, only one had very high levels of community risk score completion.

### Reach – comparison of recruitment into the intervention

#### How many people were recruited?

People could either be referred into a telephone DPP or an exercise DPP, and could later attend both services. Of the patients referred to the telephone DPP, 169 were recruited to a randomised controlled trial and are not included in this analysis. Referrals to the DPPs and the numbers who started on the programmes are summarised in Tables 6 and 7. During the research period, 724 people were referred to the telephone DPP and 139 were referred to the exercise DPP. There was some overlap in these numbers, with 81 people attending both DPP services. A total of 782 people were referred into DPP services.

**Table 6:** Telephone DPP: number of patient referrals, starts and completions, by referral route

Source of referral	Referred	Started (% of referred)	Completed (% of started)
GP nurse facilitator	633	288 (45%)	212 (74%)
Other GPs	55	38 (69%)	27 (71%)
Community	36	8 (22%)	4 (50%)
Total	724	334 (46%)	243^a^ (73%)

^a^2 patients were still in service

**Table 7:** Exercise DPP: number of patient referrals, starts and completions, by referral route

Source of referral	Referred	Started (% of referred)	Completed (% of started)
GP nurse facilitator	24	17 (71%)	14 (82%)
Other GPs	3	3 (100%)	2 (67%)
Community	22	12 (55%)	9 (75%)
Telephone DPP	90	63 (70%)	55 (87%)
Total	139	95 (68%)	80 (84%)

Once referred, 334 patients (46% or referrals) started the telephone DPP, by completing the first call; 65 patients (68% of referrals) started the exercise DPP, indicating a higher take-up rate for the exercise programme.

#### Which referral route was most effective in recruitment of eligible participants?

In the telephone DPP service, 87% of referrals came from the 16 general practices that had the additional support of a nurse facilitator, 8% from other 30 GP practices and 5% were community referrals. The conversion rate from making the referral to programme starts was 69% in the unsupported GP practices, 45% in the supported practices and 22% in the community referrals. (Table 6) This indicates that the support of the nurse facilitator was the most effective method of generating referrals to the telephone service, although a higher proportion of the referrals were converted into starts on the unsupported GP route.

When the exercise DPP service was set up, referrals could only be made by the telephone DPP provider, so it is unsurprising that 65% of referrals came from that route, 16% were community referrals, 17% from supported GPs and 2% from other GPs. The conversion rate from making the referral to actual programme starts was 71% in the supported GP practices, 100% in the unsupported practices, 55% in the community referrals and 70% in referrals from the telephone DPP. Community referral was therefore least effective in the conversion of referrals to programme starts.

### Adoption and implementation

We present the results for Adoption and Implementation together, as both were explored through the qualitative data collection and analysis, and interpreted using the NPT framework.

The most prominent themes in the data concerned:

- Perceived value of the new referral methods
- Tensions in regard to inter-organisational working
- Discrepancies between the service leads focus on targeting and how this was described by front line workers for the community route
- The need for direct communication between the organisations to resolve process issues

The above themes are reflected in Column 3 of Tables 3 and 4. Further analysis supported by NPT (Column 4 of Tables 3 and 4) enabled us to:

1. More explicitly consider coherence between participants in terms of understanding and valuing the work processes required, which consequently directly impacted on cognitive participation and collective action.
2. Consider interactions between the themes, with coherence and reflexive monitoring appearing to underpin cognitive participation and collective action, and the implications of this for future pilots (Implementation as hindered by lack of engagement with the processes, and reflective feedback between agencies being key to resolving such issues.)

In the primary care route (Table 3), the explicit acknowledgement by all actors that practices themselves were under-resourced to perform the work (*coherence*) and consequently the need for additional support specifically from the Nurse Facilitator (*cognitive participation*) meant that there was agreement about how the work should be done (*collective action*). The primary care route operated within the established clinical ecosystem, making the referral to the telephone service easier, and the Nurse Facilitator’s direct role in the telephone service itself also increased trust in the process. This contrasts with the community referral (Table 4), where there was a lack of consensus (*coherence*) on the need for the separate organisations to work jointly (*cognitive participation*), hindering integrated working (collective action). Referral into the telephone service was further complicated by being outside typical clinical systems. This directly undermined ‘*Reach’*, as it led to delays in referrals being accepted and as the focus on targeting high risk communities was lost. In both cases, the importance of reflexive action was evident with opportunities for feedback and revision of processes considered crucial to improving delivery.

However, it was apparent from the interviews with primary care that the high coherence could be negatively impacted by two particular changes to the process. Firstly, practices which did not receive full support (in the form of clinic visits) were less likely to see the route as beneficial, which is consistent with the notion that this coherence was based on the perceived need for greater support and recognition that practices themselves were under-resourced. Secondly, it emerged during the interviews that possible changes to the telephone service itself may impact on ‘buy in’. Over the course of the pilot, the telephone service moved from providing initial contacts with a Specialist Diabetes Nurse to providing all contacts through Health Support Workers. Some primary care participants suggested that this would reduce their willingness to refer given that they perceived the benefit for patients as being the contact with specialists which they otherwise would not receive. This further demonstrates the importance of coherence (in this case, the perception of additional value of the referral route beyond what practices already offered) as a key component in engagement.

NPT constructs are not intended to be linear, but interact dynamically. The data demonstrate however that without the ‘bedrock’ of coherence, cognitive participation and subsequently collective action are limited. Reflexive action emerged as essential, particularly in the community referral route, for providing the opportunity for stakeholders to dynamically revise processes and increase understanding of the different roles and ways of working.

### Maintenance

#### Individual: which factors are associated with adherence to treatment – does referral route impact on this?

We aimed to examine maintenance at both an individual and organisational level. Individually, we were interested in whether the different referral routes had different impacts on individual patients sustaining their engagement with the DPP interventions, summarised in Tables 6 and 7. Overall, DPP retention rates were high: patient who started the programme often stayed to the end. On the telephone DPP, 243 people (73% of starters) completed the programme. On the exercise DPP, 80 people (84% of starters) completed the programme. Completion rates on both programmes were lower for patients from the community referral route (50% in the telephone DPP and 75% in the exercise DPP), but this is based on small numbers of community referrals, so the result must be treated cautiously.

The qualitative data also provides insight into the processes likely to hinder or support sustainability of the interventions. Beyond initial resources (actors with appropriate expertise in clinical or community settings) and initial communications to establish a shared understanding of the need for the interventions, there is a need for sustained, supported interaction to enable process issues to be resolved. Adaptations over the course of delivery also need to be assessed for their impact on initial buy-in, as in the case (discussed in the previous section) of potential changes to which staff delivered the primary care intervention.

## Discussion

### Summary of findings

We used the RE-AIM framework to synthesise quantitative and qualitative evaluation data to inform understanding of delivery of pilot diabetes prevention services in the North of England. We summarise the key findings relating to Reach, Adoption, Implementation and Maintenance in Table 8. We then discuss the implications for policy and practice, with particular consideration of how interventions to improve Reach can anticipate challenges to Adoption and Implementation. Finally we report the limitations of the current study.

**Table 8:** Summary of key findings

RE-AIM Dimension	Key Findings
Reach	• The community campaign completed diabetes risk scores with 1162 people, and blood tests with 746 people, of which 71 were diagnosed with NDH and 66 (6%) were referred to a local DPP. The conversion rate was disappointing, suggesting that the community campaign was not particularly effective. • There were 883 referrals to the DPP from primary care A nurse facilitator undertook electronic searches and/or clinics in 16 practices and thisresulted in the referral of referred 774 (88%) patients to the DPP. The remaining 109 (12%) were referred from the 30 practices without support from the nurse facilitator. This suggests that the addition of the nurse facilitator was effective in producing more referrals. • Within thecommunity referral route, of the completed diabetes risk scores, 46% were with people over 70, 65% were for women, 7% were for someone from an ethnic minority and rates of completion were higher in the least deprived wards and those with lower rates of diabetes. This suggests that further targeting to high risk groups would be beneficial. • The community campaign led to 8 people starting the telephone DPP (22% of those referred). The facilitated GP route (16 practices) led to 288 people starting the telephone DPP (45% of those referred). The GPs without extra facilitation (30 practices) led to 3 people starting the telephone DPP (100% of those referred).
Effectiveness	*Not assessed in this study.*
Adoption	• Adoption of the intervention itself was strongly supported by the professionals involved in delivery, with consensus around the need for additional resource to support identification in primary care, and the need for community-focused organisations to expand identification beyond clinical settings. • However, in the community service, a lack of buy-in to the need for collaborative working hindered inter-agency collaboration in the early stages of delivery.
Implementation	• The facilitated GP route was comparatively easier to implement, with the role of the nurse facilitator well understood and integrated into existing processes. The community services, due to lack of consensus around the value and processes of collaborative working, did not work in the integrated way intended. Resulting pressures impacted Reach, as there was a lack of fidelity to the intended focus on high-risk patient populations. • Adaptations over time had the potential to impact both routes, both positively and negatively. In the community services, collaboration was enhanced through deliberate efforts to improve inter-agency working. In the GP route, changes to staff involved may undermine trust in the process.
Maintenance	Once people had started in a DPP programme, the retention rates were fairly high, with 73% of people completing the telephone DPP and 84% completing the exercise DPP. Retention rates were lower among the community referrals (50% in the telephone DPP and 75% in the exercise DPP), but this is based on small numbers of community referrals, so the result must be treated cautiously.

### Implications for Policy & Practice

Internationally within the USA, Finland, China and India [19–22] large clinical trial-based lifestyle focussed interventions have shown delays to, or prevention of progression to, T2DM, in a significant proportion of patients. Beyond demonstrating the effectiveness of such interventions, it is now necessary to identify the most effective ways to deliver diabetes prevention services. The present findings give insight into the organisational and delivery challenges that are likely to be encountered in the UK and beyond. Specifically, meeting public health challenges will increasingly require collaborative effort between multiple organisations, and particularly joint working across clinical and non-clinical settings. Partnership working is one the six components of effective public health interventions [23], and the present study exposes barriers to achieving effective collaboration in public health contexts. Collaboration efforts can be undermined by tensions between organisations, particularly in the context of reduced funding and between clinical and non-clinical services. These collaborations themselves may be the ‘new ways of working’ that organisations struggle to implement, as opposed to adopting a new intervention itself. Our findings are consistent with a recent evaluation of diabetes prevention in the UK which found that understanding role allocations and clarity in procedures was essential to maximising referral pathways [24].

The findings do however indicate ways in which these challenges can be addressed. Firstly, supporting understanding of the added value of collaboration and appreciation of the benefits of joint working is essential from the outset, and needs to be communicated to all levels of the organisation. Secondly, providing mechanisms and spaces for direct reflexive feedback is vital. The data demonstrate that effective collaboration requires suitable development time to enable cycles of reflection and shared learning to take place and impact on delivery. As well as overcoming practical barriers, for example around information sharing, this can also be necessary to achieve shared understanding of respective roles and contributions, which, in the present study, proved fundamental to effective partnership working. NPT has recently been expanded to explicitly focus on change over time [25], and elsewhere we have highlighted the importance of describing how interventions change over time [26]. Our findings demonstrate that implementation effects need to be understood as dynamic, to capture both unintended impacts (delays in referral between agencies) and active efforts (joint meetings to resolve issues) that contribute to implementation success.

There is a recognised need for public health programmes to reach beyond clinical settings. Primary care will always be an important source of referrals, and it is comparatively easier within general practices to search for eligible patients (using information about risk already available) and approach them about referral. Community referral faces the far greater challenge of how best to identify people in a general population who might be at risk of diabetes with no prior risk information, test their eligibility and persuade them of the need to attend a prevention programme. The present study has identified specific issues that will need to be addressed to for community services to rise to this challenge. Firstly, targeting of high-risk populations will be essential both to address unmet need in such groups and also to most efficiently use resources and ensure high conversion rates. Programmes which have focused on high-risk populations have found community screening to be effective [27]. Secondly, the difficulties of referral between clinical and non-clinical settings must be recognised and addressed. Data collection and integration can present barriers to diabetes prevention efforts [20], and the present study demonstrates that this problem may be exacerbated when non-clinical services are involved.

The qualitative findings demonstrated a failure to maintain a focus on at-risk groups, and the quantitative results suggest that further targeting to high risk groups could be considered. However, it is important to note when interpreting the data on Reach that we do not have data on the number of approaches made: it is possible that engagement activity was targeted towards those most at risk, and staff found it more challenging to persuade people to complete risk scores.

## Limitations

The services involved in referral had commissioned the service themselves so the study cannot shed light on differences in adoption between organisations, for example understanding why some providers would choose not to participate. The NPT analysis nevertheless demonstrated that the complexity of adoption in practice goes beyond organisations opting in or out of delivery, but includes which elements of an intervention can be engaged with, and engagement between participating organisations.

The topic guides were focused specifically on questions of interest to the study funders, which may have restricted the scope of the interviews and be responsible for the framework capturing all the participant data (as issues outside of this were not elicited in the interview). However, we used the topic guides in a semi-structured manner and explicitly asked participants to discuss issues they felt were important and raise issues that we may have missed. The breadth of the sample, and the multiple interviewers, again added diversity to the process. Rather than being the result of restriction, the ‘fit’ between the data and the framework may reflect the value of coproducing the topic guides with stakeholders, who were able to identify issues that had arisen or were likely to be important. Furthermore, the results were consistent with NPT, supporting the generalisability of the findings beyond this specific context.

The quantitative analysis is based on observational data, and caution is needed in interpretation of differences between the different referral routes and between the two DPP service providers, particularly given that we were unable to perform statistical comparisons. The three observational datasets were collected by three service providers, and it was not possible to match them together: we have tried to avoid any double counting, or at least report where it may occur, but this may not be exact.

We did not address the Effectiveness of the programme, and although we have provided reflections from the data on Maintenance, it should be acknowledged that the study was of a time-limited pilot programme. Given the impact of changes to ways of working over time, the study demonstrates the need for longer-term evaluation to capture the dynamic interplay of Implementation and Adoption on Reach and to fully consider whether interventions to enhance Reach have impacts on Effectiveness.

## Conclusions

Diabetes prevention is a public health priority, and there is a recognised need to tackle population health problems beyond clinical settings. The study demonstrates that this challenge should not be underestimated, and an explicit focus on collaborative working and integration between community and clinical services will be required if such initiatives are to contribute effectively to reducing diabetes risk. The RE-AIM framework provided a valuable structure for reporting key learning around both the process and outcomes of pilot diabetes prevention services. The study demonstrates the value of using a holistic framework to capture the key components of a population health service intervention and also to understand the interaction of such components over time. The nested NPT analysis underscores the importance of understanding the dynamic processes of delivery in practice. The analysis demonstrates the importance of mixed-methods evaluation, with qualitative data providing a sophisticated understanding of Adoption and Implementation to complement quantitative evaluation of Reach.

## Additional file

Additional file 1: Knowles Appendix Topic Guides. Topic Guides used in the interview study with Key Informants, Community Services and Primary Care. (DOCX 16 kb)

## Abbreviations

BME: Black and Minority Ethnic
DPP: Diabetes prevention programme
GP: General Practice
IFG: Iimpaired fasting glucose
IGR: Impaired glucose regulation
IGT: Impaired glucose tolerance
NPT: Normalisation process theory
RE-AIM: Reach, effectiveness, adoption, implementation and maintenance
T2DM: Type 2 diabetes mellitus
