# Supplementary material for: Referral of patients to diabetes prevention programmes from community campaigns and general practices: mixed-method evaluation using the RE-AIM framework and Normalisation Process Theory
Source: BMC Health Serv Res. 2019 May 22;19:321. doi: 10.1186/s12913-019-4139-5 (PMC6530123; doi:10.1186/s12913-019-4139-5)
Supplement: Supplementary file 1 — Knowles Appendix Topic Guides. Topic Guides used in the interview study with Key Informants, Community Services and Primary Care. (DOCX 16 kb) [file 12913_2019_4139_MOESM1_ESM.docx]

**Topic Guide - Key Informants**

**Describing the service:**

Could you describe the different referral routes into the diabetes prevention programme(DPP)?

Why were those routes chosen? What were the aims or hoped-for impacts?

Who organised them? How were they commissioned?

Were they based on prior way of working or existed evidence?

**Delivery:**

***Primary Care:***

Who is involved in organising and conducting primary care referral? (Probes: explore professional background and training, dedicated resource)

***Community referral:***

Who is involved in organising and conducting the community referral? (Probes: explore different professional or nonprofessional backgrounds, communication between the community services)

**Acceptability:**

***Primary Care:***

How did primary care staff respond to the enhanced referral? Was it appreciated/seen as necessary/seen as challenging?

Would there be any issues for patients identified through this referral route?

***Community referral:***

How did the community services staff work together? Were there any challenges for staff in community referral?

Do you think the community referral route is acceptable to patients? Why/why not/for whom?

**Benefits/perceived impact:**

***Primary care:***

What do you think the advantages of the enhanced primary care referral are? (Probes: for DPP, for primary care services or staff, for patients.)

What does it achieve in comparison to routine referral?

How did you assess the impact of the service?

***Community referral:***

What do you think the advantages of the community referral are? (Probes: for the different community services, or staff, for patients.)

What does it achieve in comparison to routine referral?

How did you assess the impact of the service, or different elements of it?

**Risks/limitations:**

***Primary Care:***

Are there any limitations to the enhanced primary care referral?

Are there particular elements that were more or less successful than others?

(Probe: In terms of eligibility or engagement of patients, barriers to delivery, additional resource required (either staff time or training), disruption to existing ways of working)

***Community referral:***

Are there any limitations to the community referral?

Are there particular elements that were more or less successful than others? (Probe: in terms of community champions or neighbourhood teams, communication or data sharing between teams, eligibility or engagement of patients, barriers to delivery additional resources required (either staff time or training), disruption to existing ways of working)

**Sustainability/scale:**

***Primary care:***

Do you think the enhanced primary care referral could continue beyond the NDPP evaluation? Would this be helpful – why/why not?

What would be needed to sustain it? What would be the barriers to this happening?

What advice would you give to other primary care services, in Greater Manchester and beyond, who wanted to implement an enhanced primary care referral system?

What support is needed, from whom?

What are the main barriers to delivering the system?

For whom or where would such a system be useful, or would it not benefit some services/populations (clinical/demographic)?

***Community referral:***

Do you think the enhanced community referral could continue beyond the current evaluation? Would this be helpful – why/why not?

What would be needed to sustain it? What would be the barriers to this happening?

What advice would you give to other primary care services, in Greater Manchester and beyond, who wanted to implement a community referral system?

What support is needed, from whom?

What are the main barriers to delivering the system?

For whom or where would such a system be useful, or would it not benefit some services/populations (clinical/demographic)?

**Community Referral – Community Service One**

**Describing the service/Delivery:**

Could you describe how your team identifies and refers patients into the DPP?

Why were those methods chosen? What were the aims or hoped-for impacts?

Who is involved in organising and delivering them?

Were they based on a prior way of working or existing evidence?

**Acceptability:**

How did you find the process of identifying patients? How did you find the process of receiving referrals from the other community service?

How did you find the process of referring patients to the DPP?

Do you think the way you were identifying and referring patients is acceptable for the patients themselves? Why/why not/for whom?

**Benefits/perceived impact:**

What do you think the advantages of the community referral are? (Probes: for the DPP, for services or staff, for patients.)

What does it achieve in comparison to routine referral?

How did you assess the impact of the service, or different elements of it?

**Risks/limitations:**

Are there any limitations to the community referral or things you would do differently?

Are there particular elements that were more or less successful than others? (Probe: in terms of community champions or neighbourhood teams, communication or data sharing between teams, eligibility or engagement of patients, barriers to delivery additional resources required (either staff time or training), disruption to existing ways of working)

**Sustainability/scale:**

Do you think the enhanced community referral could continue beyond the NDPP evaluation? Would this be helpful – why/why not?

What would be needed to sustain it? What would be the barriers to this happening?

What advice would you give to other primary care services, in Greater Manchester and beyond, who wanted to implement a community referral system?

What support is needed, from whom? What professional support is needed, or voluntary sector expertise?

What are the main barriers to delivering the system?

For whom or where would such a system be useful, or would it not benefit some services/populations (clinical/demographic)?

**Community Referral – Community Service Two**

**Describing the service/Delivery:**

Could you describe how your service identifies and refers patients into the DPP?

Why were those methods chosen? What were the aims or hoped-for impacts?

Who is involved in organising and delivering them?

Were they based on a prior way of working or existing evidence?

**Acceptability:**

How did you find the process of identifying patients?

How did you find the process of referring patients?

Do you think the way you were identifying and referring patients is acceptable for the patients themselves? Why/why not/for whom?

**Benefits/perceived impact:**

What do you think the advantages of the community referral are? (Probes: for the DPP, for the community services or staff, for patients.)

What does it achieve in comparison to routine referral?

How did you assess the impact of the service, or different elements of it?

**Risks/limitations:**

Are there any limitations to the community referral or things you would do differently?

Are there particular elements that were more or less successful than others? (Probe: in terms of community champions or neighbourhood teams, communication or data sharing between teams, eligibility or engagement of patients, barriers to delivery additional resources required (either staff time or training), disruption to existing ways of working)

**Sustainability/scale:**

Do you think the enhanced community referral could continue beyond the NDPP evaluation? Would this be helpful – why/why not?

What would be needed to sustain it? What would be the barriers to this happening?

What advice would you give to other primary care services, in Greater Manchester and beyond, who wanted to implement a community referral system?

What support is needed, from whom? What professional support is needed, or voluntary sector expertise?

What are the main barriers to delivering the system?

For whom or where would such a system be useful, or would it not benefit some services/populations (clinical/demographic)?

**Topic Guide – Primary Care**

**Describing the service/delivery:**

Could you describe the enhanced primary care referral route into the DPP?

Why was the enhanced route introduced? What were the aims or hoped-for impacts?

Who organised it and funded it?

Was it based on a prior way of working or existed evidence?

Who was involved in organising and conducting primary care referral? (Probes: explore professional background and training, dedicated resource, additional practice staff involvement)

**Acceptability:**

How did you find the enhanced referral? Do you think it was helpful?

Do you think it was acceptable to patients themselves? Would there be any issues for patients identified through this referral route?

**Benefits/perceived impact:**

What do you think the advantages of the enhanced primary care referral are? (Probes: for the DPP, for primary care services or staff, for patients.)

What does it achieve in comparison to routine referral?

How did you assess the impact of the service?

**Risks/limitations:**

Are there any limitations to the enhanced primary care referral?

Are there particular elements that were more or less successful than others?

(Probe: In terms of eligibility or engagement of patients, barriers to delivery, additional resource required (either staff time or training), disruption to existing ways of working)

**Sustainability/scale:**

Do you think the enhanced primary care referral could continue beyond the NDPP evaluation? Would this be helpful – why/why not?

What would be needed to sustain it? What would be the barriers to this happening?

What advice would you give to other practices, in Greater Manchester and beyond, who wanted to implement an enhanced primary care referral system?

What support is needed, from whom?

What are the main barriers to delivering the system?

For whom or where would such a system be useful, or would it not benefit some services/populations (clinical/demographic)?
